# Bayesian Mixture Model Analysis for Detecting Differentially Expressed Genes

**DOI:** 10.1155/2008/892927

**Published:** 2008-03-25

**Authors:** Zhenyu Jia, Shizhong Xu

**Affiliations:** ^1^Department of Pathology & Laboratory Medicine, University of California, Irvine, CA 92697, USA; ^2^Department of Botany and Plant Sciences, University of California, Riverside, CA 92521, USA

## Abstract

Control-treatment design is widely used in microarray gene expression experiments. The purpose of such a design is to detect genes that express differentially between the control and the treatment. Many statistical procedures have been developed to detect differentially expressed genes, but all have pros and cons and room is still open for improvement. In this study, we propose a Bayesian mixture model approach to classifying genes into one of three clusters, corresponding to clusters of downregulated, neutral, and upregulated genes, respectively. The Bayesian method is implemented via the Markov chain Monte Carlo (MCMC) algorithm. The cluster means of down- and upregulated genes are sampled from truncated normal distributions whereas the cluster mean of the neutral genes is set to zero. Using simulated data as well as data from a real microarray experiment, we demonstrate that the new method outperforms all methods commonly used in differential expression analysis.

## 1. INTRODUCTION

Current microarray technology allows us to measure the expression of thousands of genes simultaneously in a small chip. Many advanced statistical methods have been developed to analyze data generated from this technology. For example, a Bayesian model has been proposed to normalize gene expression data and select genes that are differentially expressed [1]. Jörnsten et al. [2] developed a Bayesian method to impute missing values prior to any statistical analysis. Efron et al. [3], Broët et al. [4], Edwards et al. [5], Do et al. [6] used a Bayesian approach to identify differentially expressed genes. Methods of [7–9] are examples for Bayesian clustering analysis.

In this study, we focus on detecting differentially expressed genes in two-sampled designs, in which only two conditions (control and treatment) are examined and each condition is replicated several times. The data are assumed to have been properly imputed and normalized as needed. Much effort has been made on this study to detect genes whose expression levels respond to the treatment.

Numerous methods have been suggested based on the *P*-values of a test statistic. The *P*-values are obtained from separate tests and reported for all genes [10, 11]. Cui and Churchill [12] reviewed the test statistics for differential expression for microarray experiments. The gene-specific summary statistic provides a basis for the rank ordering of genes and the creation of a short list of genes declared as differentially expressed. However, genes are studied separately in the sense that calculating the test statistic for one gene does not use information from other genes. In limma (linear model for microarray data), Smyth [13] cleverly borrowed information from the ensemble of genes to make inference for individual gene based on the moderate *t*-statistic. Some other researchers also took advantages of shared information by examining data jointly. Efron et al. [3] proposed a mixture model methodology implemented via an empirical Bayes approach. For each gene, the expression levels were mapped to a single *z*-score, which is assumed to arise from two distributions (affected and unaffected). Pan [14] used a mixture model to cluster genes based on gene specific *t*-statistic. They are examples where the idea of clustering was first used in differential expression analysis (clustering methods are usually applied to microarray data generated from multiple conditions). Similarly, the methods of [4–6, 15] all used a mapped quantity, like the *z*-score or the *t*-statistic, to classify genes into different groups. These methods are different from the proposed Bayesian mixture model in that genes were clustered based on a summary statistic for these methods while our Bayesian method clusters genes based on the pattern of expression. It is more desirable to use the expression profile than to use a summary score for cluster analysis because information is bound to be lost when mapping the expression profile into a single score. Recently, Newton et al. [16] developed a new method in which gene expression are directly used for classification. Newton et al. [16] used a hierarchical model that consists of two levels. The first level of the model describes the conditional distributions of the observed measurement of gene expression given the means of the control (*μ*_1*i*_) and the treatment (*μ*_2*i*_), where *i* indexes the gene. The second level describes the distribution of *μ*_1*i*_ and *μ*_2*i*_ jointly by a mixture of three multivariate distributions with constrained parameters of *μ*_1_ > *μ*_2_, *μ*_1_ = *μ*_2_, and *μ*_1_ < *μ*_2_. Genes become linked by virtue of having *μ*_1*i*_ and *μ*_2*i*_ drawn from a common distribution. Parameter estimation was accomplished via the Expectation-Maximization (EM) algorithm [17].

For the Bayesian clustering method presented in this study, the observed gene expression levels are described by a regression model as done by Gusnanto et al. [15]. For each gene, the irrelevant intercept is removed by a special normalization scheme. The slope of the regression represents the difference of expression under the two conditions. The Bayesian method is implemented via the Markov chain Monte Carlo (MCMC) algorithm. The regression coefficient of each gene is assumed to be sampled from a mixture of three normal distributions with constrained parameters. The three distributions are *N*(*β_k_*, *ν_k_*) for *k* = 1, 2, 3, where *β*_1_ < 0, *β*_2_ = 0, and *β*_3_ > 0 are the constrained parameters. The proposed new method actually turns the problem of a complicated multivariate mixture distribution of Newton et al. [16] into that of a univariate mixture distribution.

The new Bayesian method proposed in this study (Method I) is compared to five different methods that are commonly used in differential expression analysis. These four methods, called Methods II, III, IV, V, and VI respectively, are described below. Method II is the regularized *t*-test (SAM, Tusher et al. [10]), in which a *t*-like score is calculated for each gene. Genes with scores greater than an adjusted threshold are said to have altered expressions under the treatment. Permutations are used to calculate the false discovery rate (FDR, Benjamini and Liu [18]). Method III is the model-based cluster analysis of Pan [14] where the variable used for clustering is the *t*-test statistic. Genes that are assigned into a cluster with a mean *t* value significantly different from zero are declared to be differentially expressed. Method IV is the hierarchical mixture model method of Newton et al. [16], where the expected expression values of the *i*th gene under the control (*μ*_1*i*_) and the treatment (*μ*_2*i*_) are assumed to arise from a mixture of three distributions. Clustering is actually conducted based on the latent parameters (*μ*_1*i*_ and *μ*_2*i*_). The cluster of genes with *μ*_1*i*_ ≠ *μ*_2*i*_ are declared as differentially expression genes. A short list of significant genes is created based on FDR at 0.05. Method V is the linear model and empirical Bayes method (limma) of Smyth [13]. In limma, a hierarchical linear model is used to describe the gene expression levels. A moderated *t*-statistic, with extra information borrowed from other genes, is calculated for each gene. Adjusted *P*-values are calculated for genes based on the moderated *t*-statistics to achieve FDR control. Method VI is the mixture mixed model analysis of Gusnanto et al. [15], where a similar linear model is used to describe gene expressions. However, the variable used for clustering is the mean difference between the treatment and the control. The mathematical differences between the proposed method and the four methods compared will be evident after the new method is introduced. Advantages of the new method over the four methods will be demonstrated in Section 3 and further addressed in the Section 4.

## 2. METHODS

### 2.1. Linear model

Let *Y_ij_* (*i* = 1,…, *m* and *j* = 1,…*N*) be the log transformed expression level for gene *i* from chip *j*, where *m* is the total number of genes and *N* is the total number of chips. Let *X_j_* be a dichotomous variable indicating the status of the chip with *X*_j_ = 0 for the control and *X*_j_ = 1 for the treatment. We use the following regression model to describe *Y_ij_*:

(1) $$Y_{ij}=\alpha_{i}+X_{j}\gamma_{i}+\varepsilon_{ij},$$

where *α_i_* is the intercept of the regression model,*γ*_i_ is the regression coefficient and *ε*_ij_ is the residual error. Note that the regression coefficient represents the difference in gene expression between the control and the treatment (*μ*_2_ − *μ*_1_). A large *γ*_i_ implies that gene *i* is differentially expressed. The intercept *α_i_* represents the mean expression level of gene *i* in the control (*μ*_1_). The intercept *α*_i_ is irrelevant to the differential expression analysis and thus should be removed from the model (see next section for detail). The regression coefficient *γ*_i_ is assumed to be sampled from one of three normal distributions, corresponding to the downregulated genes, the neutral genes, and the upregulated genes, respectively. The residual error is assumed to be *N*(0, *σ* ^2^) distributed. In matrix notation, model (1) can be expressed as

(2) $$Y_{i}=W\alpha_{i}+X\gamma_{i}+\varepsilon_{i},$$

where *Y_i_* = {*Y*_ij_}_*j* = 1_ is a column vector for the expressions of gene *i* across all samples, *X* = {*X*_j_}_*j* = 1_^*N*^ is the incidence matrix (a column vector) for the linear model, *ε_i_* = {*ε_ij_*}_*j* = 1_ *^N^* is a vector for the residual errors, and *W* is an *N* × 1 unity vector, that is, a vector of 1 for all elements.

### 2.2. Normalization

As stated earlier, *α_i_* is irrelevant to the differential expression analysis. We propose to remove the effect of *α* by using a linear contrast. This process, which is called normalization, is similar to the way of removing the fixed effects in the restricted maximum likelihood (REML) method for variance component analysis [19]. For each gene, we subtract the observed expression level by the average level across all chips, that is,

(3) $$Y_{i}^{*}=Y_{i}-W{\bar{Y}}_{i,}$$

where $\bar{Y}$ = *N* ^−1^∑_*j* = 1_ *^N^* *Y_ij_* is the mean expression for gene *i* across all the chips. The mean expression can also be expressed in matrix notation as

(4) $${\overset{¯}{Y}}_{i.}=(W^{T}W{)}^{-1}W^{T}Y_{i.}.$$

Therefore, the normalized expression is described as a linear combination of the original expressions

(5) $$Y_{i}^{*}=[I-W{\left(W^{T}W\right)}^{-1}W^{T}]Y_{i}=LY_{i},$$

where

(6) $$L=I-W{\left(W^{T}W\right)}^{-1}W^{T}.$$

We can prove that *LW* = 0. Therefore,

(7) $$Y_{i}^{*}=LY_{i}=LX\gamma_{i}+L\varepsilon_{i.}$$

Let X* = *LX* and *ε*_i_* = *Lε_i_*, we have

(8) $$Y_{i}^{*}=X^{*}\gamma_{i}+\varepsilon_{i}^{*}.$$

Now the intercept has been removed by using linear contrasts *LY_i_* in place of the original *Y_i_*. The residual variance-covariance matrix of the linear contrasts is Var(*ε_i_* *) = *LL* ^T^ *σ* ^2^. Note that the rank of matrix *LL* ^T^ is *N* − *r*(*W*) = *N* − 1, indicating that the inverse of matrix *LL* ^T^ does not exist. The model-based cluster analysis, however, requires the inverse. Therefore, we delete the last row of matrix *L* to form a new (*N* − 1) × *N* matrix, called L*, to build the linear contrasts. This treatment is equivalent to deleting the last element of *Y_i_* *, that is, the dimension of *Y_i_* * is (*N* − 1) × 1. The dimensions of matrix X* and *ε_i_* * also change into (*N* − 1) × 1 accordingly. Let *R* = L*(L*)^T^ be an (*N* − 1) × (*N* − 1) matrix of full rank. We now have Var(*ε_i_* *) = *R^σ2^*, which will be used in the model-based cluster analysis.

To simplify the notation, let us define *y_i_* = *Y_i_* *, *x* = X* and $\epsilon_{i}=\varepsilon_{i}^{*}$ all with a dimension of (*N* − 1) × 1. The new model with the intercept removed becomes

(9) $$y_{i}=x\gamma_{i}+\epsilon_{i}.$$

Note that the special way of normalization described above only applies to linear effects. Different methods should be used for adjusting nonlinear effects. In subsequent analysis, we assume that normalization via linear contrasts has been conducted and thus model (9) will be the basis for parameter estimation and statistical inference.

### 2.3. Mixture model and the Bayesian setup

Conditional on *γ_i_*, the probability density of *γ_i_* is

(10) $$p(y_{i}\;|\;\gamma_{i},\;\sigma^{2})=\phi_{n}(y_{i};\;x\gamma_{i},\;R\sigma^{2}),$$

where *n* = *N* − 1, *ϕ_n_* (*yi*; *xγ_i_*, *Rσ* ^2^) is the *n*-dimensional normal density of variable *y_i_* with mean *xγ_i_* and variance-covariance matrix *Rσ* ^2^. The gene-specific effect *γ_i_* is assumed to be sampled from one of three normal distributions (clusters), *N*(*β_k_*, *ν_k_*) for *k* = 1, 2, 3. We define variable *z_i_* as the cluster assignment of gene *i*, where *z_i_* = *k* if gene *i* comes from cluster *k*. For notational simplicity, we define *η*(*z_i_*, *k*) for *k* = 1, 2, 3 as a redundant variable to *z_i_*, where *η*(*z_i_*, *k*) = 1 if *z_i_* = *k* and *η*(*z_i_*, *k*) = 0 otherwise. Let *η*(*z_i_*) = {*η*(*z_i_*, *k*)}_*k* = 1_^3^ for *η*(*z_i_*, *k*) = 0, 1 and ∑_*k* = 1_^3^ *η*(*z_i_*, *k*) = 1. The new variable *η*(*z_i_*) will be used in place of *z_i_* in subsequent derivation. Let *β* = {*β_k_*}_*k* = 1_^3^ and *ν* = {*ν_k_*}_*k* = 1_^3^. The density of the mixture distribution of *γ_i_* is

(11) $$p(\gamma_{i}\;|\;\eta (z_{i}),\;\beta ,\;\nu )=\overset{3}{\underset{k=1}{\sum }}\eta (z_{i},\;k)\phi_{1}(\gamma_{i};\;\beta_{k},\;\nu_{k}).$$

Note that variable *η*(*z_i_*, *k*) is unknown and it is treated as missing value. In fact, inferring the probability distribution of *η*(*z_i_*, *k*) is the main purpose of the proposed mixture model analysis. Define *π* = {*π_k_*}_*k* = 1_^3^ as the mixing proportions of the three components for *π_k_* ≥ 0 and ∑_*k* = 1_^3^ *π_k_* = 1. The distribution of *η*(*z_i_*) is multinomial with one observation,

(12) $$p(\eta (z_{i})\;|\;\pi )=\overset{3}{\underset{k=1}{\prod }}\pi_{k}^{\eta (z_{i},k)}.$$

We have introduced the probability densities of the data, the regression coefficients, and the missing values for a single gene. We now combine the densities of individual genes to form the joint density of all genes. The probability density of the data *y* = {*y_i_*}_*i* = 1_^m^ is

(13) $$p(y\;|\;\gamma ,\;\sigma^{2})=\overset{m}{\underset{i=1}{\prod }}p(y_{i}\;|\;\gamma_{i},\;\sigma^{2}).$$

The density of *γ* = {*γ_i_*}_*i* = 1_^m^ is

(14) $$p(\gamma \;|\;\eta ,\;\beta ,\;\nu )=\overset{m}{\underset{i=1}{\prod }}p(\gamma_{i}\;|\;\eta (z_{i}),\;\beta ,\;\nu ).$$

The density of *η* = {*η*(*z_i_*)}_*i* = 1_^m^ is

(15) $$p(\eta \;|\;\pi )=\overset{3}{\underset{k=1}{\prod }}\pi_{k}^{m_{k}},$$

where *m_k_* = ∑_*i* = 1_^m^ *η*(*z_i_*, *k*) for ∑_*k* = 1_^3^ *m_k_* = *m*. The joint density of (*y, γ, η*) is

(16) $$p(y,\;\gamma ,\;\eta \;|\;\theta )=p(y\;|\;\gamma ,\;\sigma^{2})p(\gamma \;|\;\eta ,\;\beta ,\;\nu )p(\eta \;|\;\pi ),$$

where *θ* = (*σ* ^2^, *β*, *π*, *ν*) is the parameter vector.

The next step of the analysis is to find a suitable prior for *θ*. The following vague prior is chosen for each of the variance components, *σ* ^2^ *∼*Inv − *χ* ^2^(0, 0) and *ν_k_* *∼*Inv − *χ* ^2^(0, 0) for *k* = 1, 2, 3. They are called the scaled inverse chi-square distributions with zero degrees of freedom and zero-scale parameter. The actual forms of the priors are *p*(*σ* ^2^) = 1/*σ* ^2^ and *p*(*ν_k_*) = 1/*ν_k_* for *k* = 1, 2, 3. The scaled inverse chi-square distribution is conjugate, and thus the posterior distribution of the variance is also scaled inverse chi-square. The three clusters of genes are distinguished by the overall patterns of responses to the treatment. The first cluster consists of all the downregulated genes, the second cluster represents all the neutral genes, and the third cluster contains all the upregulated genes. The characteristics of the three clusters can be represented by enforcing the following constraints on the means of the three clusters: *β*_1_ < 0, *β*_2_ = 0, and *β*_3_ > 0. Therefore, the prior distributions of the means of the three clusters are *p*(*β_k_*) = 1 (*β*_k_ ∈ Ω*_k_*). Here, we adopt a special notation to indicate that *p*(*β_k_*) = 1 if (*β_k_* ∈ Ω*_k_*) and *p*(*β_k_*) = 0 if (*β_k_* ∉ Ω*_k_*, where Ω_1_ ≡ (*β*_1_ < 0), Ω_2_ ≡ (*β*_2_ = 0), and Ω_3_ ≡ (*β*_3_ > 0). The prior for *π* is the multivariate generalization of the Beta distribution *p*(*π* ∣ *δ*) = *D*(*π* ∣ *δ*, *δ*, *δ*), called the Dirichlet distribution, where *δ* = 1 is used in this study. The joint prior for all the parameters is *p*(*θ*) = (1/*σ* ^2^)∏_*k* = 1_^3^ (1/*ν_k_*). The posterior distribution of all the unknowns *p*(*γ*, *η*, *θ* ∣ *y*) is proportional to the joint distribution of all variables,

(17) $$\begin{array}{ll} p(\gamma ,\;\eta ,\;\theta \;|\;y) & \propto p(y,\;\gamma ,\;\eta ,\;\theta )\\ & =p(y\;|\;\gamma ,\;\sigma^{2})p(\gamma \;|\;\eta )p(\eta \;|\;\pi )p(\theta ). \end{array}$$

Statistical inference of this distribution is the theme of the Bayesian analysis. There is no explicit form for the joint distribution (17). Therefore, we draw observations of the unknowns from the conditional distributions. Fortunately, with the current Bayesian setup, the conditional distribution of any single variable, given all other variables, has a known distribution. Therefore, the MCMC process can be proceeded exclusively using the Gibbs sampler.

### 2.4. Markov chain Monte Carlo

The detailed steps of the Markov chain Monte Carlo (MCMC) process are described as follows.

**Step 1.**

Set *t* = 0 and initialize all variables (*γ* ^(*t*)^, *η* ^(*t*)^, *β* ^(*t*)^, *σ* ^2^ ^(*t*)^, *ν* ^(*t*)^, *π* ^(*t*)^).

**Step 2.**

Simulate *γ_i_* ^(*t* + 1)^ from $\gamma_{i}∼N({\bar{\gamma }}_{i},\;s_{\gamma_{i}}^{2})$ conditional on *z_i_* = *k*, where

(18) $$\begin{array}{c} s_{\gamma_{i}}^{2}={\left[x^{T}R^{-1}x+\sigma^{2}/\nu_{k}\right]}^{-1}\sigma^{2}\\ {\overset{¯}{\gamma }}_{i}={\left[x^{T}R^{-1}x+\sigma^{2}/\nu_{k}\right]}^{-1}\left[x^{T}R^{-1}y+(\sigma^{2}/\nu_{k})\beta_{k}\right], \end{array}$$

where all variables in the right-hand side take the most current values. In this step, the most current values of the variables in the right-hand side are the values at iteration *t*. This statement also holds for subsequent steps, except that the most current values of the variables in the right-hand side are values at iteration *t* or *t* + 1, depending on whether the variable has been updated or not in the current sweep.

**Step 3.**

Simulate *η*(*z_i_*)^(*t* + 1)^ from

(19) $$\eta (z_{i})∼\text{Multinomial }(1,\pi_{i1},\;\pi_{i2},\;\pi_{i3}),$$

a multinomial distribution with one observation, where *π_ik_* is

(20) $$\pi_{ik}=\frac{\pi_{k}p(\gamma_{i}\;|\;\eta (z_{i},\;k),\;\beta_{k},\nu_{k})}{\sum_{k^{\prime }=1}^{3}\pi_{k^{\prime }}p(\gamma_{i}\;|\;\eta (z_{i},k^{\prime }),\;\beta_{k^{\prime }},\nu_{k^{\prime }})}.$$

**Step 4.**

Simulate *β_k_* ^(*t* + 1)^ from a truncated normal distribution

(21) $$\beta_{k}∼N({\overset{¯}{\beta }}_{k},\;s_{\beta_{k}}^{2})1(\beta_{k}\in \Omega_{k}).$$

The mean and the variance of the normal distribution are

(22) $$\begin{array}{ll} {\overset{¯}{\beta }}_{k} & =\frac{1}{\sum_{i=1}^{m}\eta (z_{i},\;k)}\overset{m}{\underset{i=1}{\sum }}\eta (z_{i},\;k)\gamma_{i},\;\\ s_{\beta_{k}}^{2} & =\frac{\nu_{k}}{\sum_{i=1}^{m}\eta (z_{i},k)}, \end{array}$$

respectively. Note that *β*_2_ = 0 is enforced and no sampling for *β*_2_ is required. The general inverse transformation method [20] is used to sample *β*_1_ and *β*_3_ from the corresponding truncated normal distributions.

**Step 5.**

Simulate *σ* ^2(*t* + 1)^ from

(23) $$\sigma^{2}∼\text{Inv}-\chi^{2}\left(mn,\;\sum {\left(y_{i}-x\gamma_{i}\right)}^{T}R^{-1}(y_{i}-x\gamma_{i})\right),$$

where *m* × *n* is the degree of freedom, and the term with summation is the scale parameter of the inverse chi-square distribution.

**Step 6.**

Simulate *ν_k_* ^(*t* + 1)^ from

(24) $$\nu_{k}∼\text{Inv}-\chi^{2}\left(m_{k},\sum \eta (z_{i},k)(\gamma_{i}-\beta_{k}{)}^{2}\right),$$

where *m_k_* = ∑_*i* = 1_^*m*^ *η*(*z_i_*, *k*).

**Step 7.**

Simulate *π* ^(*t* + 1)^ from

(25) $$\pi ∼\text{Dirichlet }(m_{1}+1,\;m_{2}+1,\;m_{3}+1).$$

So far, every variable has been updated. We then increment *t* by 1(*t* = *t* + 1) and repeat Steps 3–7 until the chain gets sufficiently long to allow reliable post-MCMC inference about the parameters of interest. Gene *i* is assigned to group *k* if $\bar{\eta }(z_{i},\;k)=\max{\left\{\bar{\eta }(z_{i},\;k^{\prime })\right\}}_{k^{\prime }=1}^{3}$ , where $\bar{\eta }(z_{i},\;k)$ is the posterior mean of *η*(*z_i_*, *k*) calculated from the posterior sample.

Schemes for sampling variables from the aforementioned distributions are discussed by Gelman et al. [21].

## 3. APPLICATIONS

### 3.1. Analysis of simulated data

**Figure 1:**
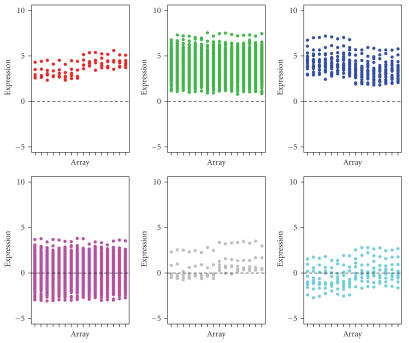
Original expression patterns for the six simulated groups of genes. In each plot, the first half represents the observed data from chips 1–8 and the second half represents the observed data from chips 9–16.

**Figure 2:**
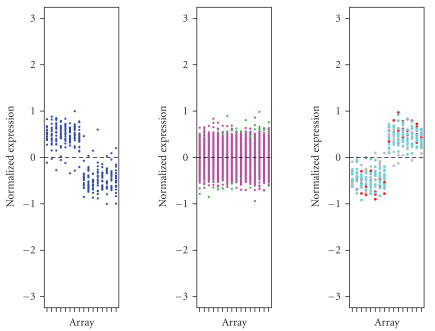
Expression patterns for the three combined clusters after normalization. See Figure 1 for the legends.

We simulated the expression of *m* = 1000 genes from six different groups on *N* = 16 microarray chips. Chips 1–8 represent the control, and chips 9–16 represent the treatment. The true parameter values of the six groups of genes are shown in Table 1. If we ignore the intercepts, the six groups of genes really come from three functional clusters: Cluster 1 represents the (*m*_1_ = 20) downregulated genes (Group 3); Cluster 2 contains the (*m*_2_ = 960) neutral genes (Groups 2 and 4); and Cluster 3 consists of the (*m*_3_ = 20) upregulated genes (Groups 1, 5, and 6). The true parameters of the original six groups and the parameters for the combined three clusters are given in Table 1. Note that there are actually three components (Group 1, 5, and 6) for the combined Cluster 3. The weighted *β* of three simulated components is treated as the true *β* for this cluster, that is, 1.2 × 0.25 + 0.8 × 0.25 + 0.9 × 0.5 = 0.95. The expression patterns are shown in Figure 1 for the original six groups and Figure 2 for the combined three clusters.

The data were analyzed using the Bayesian mixture model analysis reported in this study (Method I). We set the number of iterations equal to 12000 in the MCMC process. The results generated from the first 2000 iterations were discarded for the burn-in period. For the remaining 10000 iterations, we saved one observation in every 20 iterations to reduce series correlation between consecutive observations. Therefore, the posterior sample contained 500 observations. The posterior mean of each variable is considered as the Bayesian estimate. Hypothesis tests were only performed on the cluster means *β* rather than on individual genes. Genes assigned into a significant cluster were simultaneously declared to be differentially expressed. From Table 1, we can see that the estimated parameters agree well with the true parameters. Genes are correctly assigned into three clusters except that two genes in Cluster 2 (the neutral cluster) are incorrectly assigned into the other two clusters (down- and upregulated clusters) (see Table 2). Upon enforcing constraints on the cluster means, we are able to fix the number of clusters to three, rather than finding the optimal number of clusters using the Bayes factor or its BIC [22] approximation.

An interesting question is what would happen if the cluster means are not constrained? To answer this question, we also analyzed the simulated data with unconstrained *β*. We varied the number of clusters from 3 to 6 and found out that 4 was the optimal number of clusters (maximum value of Bayes factor). One cluster mean was close to zero. Another cluster had a negative mean. The third and fourth clusters all had positive means but the two means were almost identical. Therefore, Clusters 3 and 4 may be well considered as a single cluster. When treated as three clusters, the results were similar to those reported earlier when the cluster means were restricted (data now shown).

For comparison, the data were also analyzed using the five methods (Methods II—VI) described in Section 1. The numbers of genes classified into each of the three clusters are given in Table 2 for all the six methods, including Method I (the new method). Cutoff value 0.05 was used for gene detection in Method II and Method V to achieve FDR control. All methods have successfully found the 40 truly differentially expressed genes. However, Methods II, III, IV, V, and VI detected more false-positive genes than Method I. The model-based cluster analysis of Pan (Method III) required BIC to decide the optimal number of clusters. For simplicity of comparison, we only used the result of Method III under three clusters. We noticed that Method III always put the down- and upregulated genes into a cluster with a large variance, but placed the neutral genes into the other two cluster with small variances. The two clusters with small variances can be combined as a single cluster. Because all the methods have successfully detected the 40 nonneutral genes, none of them have suffered the Type II error. However, different methods tended to have different numbers of genes from the neutral cluster misplaced into the functional clusters. This means that different methods have different Type I errors.

Let *N*(*k*, *k*′) be the number of genes from cluster *k* assigned into cluster *k*′ for *k*, *k*′ = 1, 2, 3. Then *N*(*k*) = ∑_*k*′ = 1_^3^ *N*(*k*, *k*′) is the number of genes in cluster *k*. Recall that Clusters 1 and 3 contain differentially expressed genes and Cluster 2 is reserved for the neutral genes. The empirical Type I error is then defined as

(26) $$\text{Type I error}=\frac{N(2,1)+N(2,3)}{N(2)}.$$

The empirical Type II error is defined as

(27) $$\text{Type II error}=\frac{N(1,2)+N(3,2)}{N(1)+N(3)}.$$

The empirical power is defined as

(28) Power=1−Type II error.

The empirical Type I errors for all the five methods are listed in Table 2. Method I (the new method) have the smallest Type I error. Note that this method of validation is valid only for simulation study where the true gene assignment is known.

### 3.2. Analysis of mice data

We analyzed a cDNA microarray dataset published by Dudoit et al. [11]. The data are publicly available on the website (http://www.stat.berkeley.edu/users/terry). The data consist of expression measurements of 6342 genes from a study of lipid metabolism in mice [23]. This experiment was set up to find genes that are differentially expressed in the livers of mice with very low HDL cholesterol levels (treatment) in contrast to a group of normal inbred mice (control). The treatment group consists of eight scavenger receptor BI (SR-BI) transgenic mice and the control group consists of eight normal inbred mice.

We first analyzed the data with the Bayesian mixture model method proposed in this study (Method I). We used exactly the same setup of the MCMC as that used in the simulation experiment. The estimated parameters are given in Table 3. Only *β*_3_ (cluster mean of up-regulated genes) is significantly different from zero. The mixing proportions of the three clusters show that majority of the genes (82.46%) are neutral. Note that the estimated mixing proportions ( ${\hat{\pi }}_{1}=0.1724$ , ${\hat{\pi }}_{2}=0.8246$ , ${\hat{\pi }}_{3}=0.0030)$ are some nuisance parameters, which do not necessarily match the actual numbers of genes assigned to the three clusters (*m*_1_ = 0, *m*_2_ = 6332, *m*_3_ = 10). Report should be made based on the actual numbers of genes assigned to the clusters.

All six methods that were used to analyze the simulated data were applied here to analyze the mice data. The numbers of genes assigned to the three clusters are given in Table 4 for all the five methods. Clearly, Methods I and VI were similar to each other and Methods II, III, IV, and V placed much more genes into the nonneutral clusters, a phenomenon which has been observed early in the simulation experiment.

In the original study [23], individual *P*-values were calculated based on the *t*-test statistics for all the genes and then the adjusted *P*-values were calculated using the method described in Westfall and Young [24]. Five genes (represented by eight array elements) were detected by Callow et al. [23] as differentially expressed between the treatment and the control (see Table 5). Note that several array elements may represent the same gene. We found that gene EST AI746730 is not included in the dataset and thus was not analyzed with any of the methods (marked as NA in Table 5).

**Figure 3:**
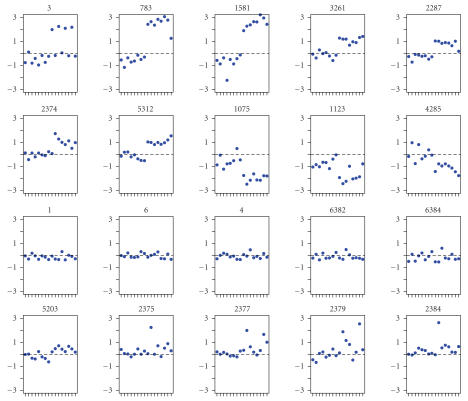
Expression patterns of some genes from the mice data. The first two rows (representing ten genes) are genes detected by all methods. The third row (five genes) are genes detected by none of the methods. The last row (five genes) are those detected by Methods II–VI but not by Methods I.

The Bayesian mixture model analysis detected 10 genes (or spots), four of which were reported in Callow et al. [23]. The expressions of the 10 genes are plotted in Figure 3 (the first two rows). Significant differences between the control and treatment groups can be easily seen. Note that element 2374 (the first plot of the second row of Figure 3) was a “blank” spot on the chip. However, it was detected by all methods due to the remarkable difference between the control and the treatment. The third row of the plots represents five randomly selected genes which were not detected by any of the methods. Genes of this kind have the same expression levels under the control and treatment. The last row of the plots (in Figure 3) shows the expression patterns of five genes that were not detected by the Bayesian mixture model analysis (Method I), but detected by the other five methods (Methods II, III, IV, V, and VI). When examining these five genes, we found that the differences between the treatment and the control are not obvious. The statistical significance may be caused entirely by the outliers. Therefore, the five methods (II, III, IV, V, and VI) are perhaps too sensitive to outliers. For example, the first plot (5203) of the last row in Figure 3 is the spot that represents *β*-globin in the dataset. We cannot tell much difference between the control and the treatment. Genes with numbers 2375, 2377, 2379, and 2384 are all “blank” spots. The observed differences between the control and the treatment are purely caused byf chance, yet these blank spots were detected by Methods II, III, IV, V, or VI. Finally, Method III [14] and Method V [13] missed gene SR-BI at array element number 3, which is known to have altered the expression between the control and the SR-BI transgenic mice.

When we examined the plots of the ten genes detected by the Bayesian mixture model analysis, we found that seven of them have increased the expression level by the treatment while three of them have decreased the expression level by the treatment. Interestingly, our method assigned the three genes with negative regression coefficients into the cluster with positive regression coefficients. If we look at the estimated parameters (Table 3) again, we realized that both Clusters 1 and 2 may be considered as the neutral cluster (mean close to zero and variance very small). The third cluster contains both the up-and downregulated genes because it has a much larger variance than the other two clusters. The same phenomenon also occurred for Method VI [15], but the issue was not discussed by Gusnanto et al. [15]. A possible explanation is that there are too few genes with negative regression coefficients, which made them hard to form a single cluster by themselves. However, why were these three genes assigned into Cluster 3 (*β* > 0) instead of Cluster 2 (*β* = 0), which is closer to the three genes in terms of the cluster mean? The reason may be that Cluster 3 has a larger variance than Cluster 2.

A separate simulation experiment has been conducted to verify the notion that *ν* plays a more important role than *β* in calculating the posterior probability of cluster assignment *π_ik_* (data not shown).

## 4. DISCUSSION

Similar to Method IV [16], our method is based on a hierarchical model in which the parameters of interest (gene effects) are further described by a mixture distribution. Clustering is made based on the parameter rather than on a summary statistic such as the *t*-like statistical score. This allows us to incorporate the error structure of the gene expression profile into the linear model (see (10)), and thus capture the most information from the data. This explains why our method is different from (or even better than) the other four methods (Methods II, III, V, and VI). But why is our method better than (or different from) Method IV? This may be contributed by the two differences between Methods I and IV. One difference is that the incorporated normalization scheme in our model allows us to deal with only the effect of differential expression (regression coefficient) whereas Method IV deals with effects of gene expressions in both the control and the treatment. In other words, we are dealing with a single variable (regression coefficient, the only parameter of interest) but Method IV deals with two variables (intercept and regression coefficients). The dimension reduction from two to one and the simplified Gaussian mixture distribution of our model may largely contribute to the higher efficiency of our method. The second difference between our method and Method IV is that we used the probabilities of cluster assignment to classify genes into three clusters, and genes assigned into the neutral cluster are excluded from the list of differentially expressed genes, but Method IV goes one step further by employing an FDR criterion to select a list of differentially expressed genes. It is obvious that the FDR generated list of genes depends largely on the subjective cutting rule set by the investigator. We consider that the extra step of FDR analysis after gene clustering is not only redundant but also leads to subjective decision for differential expression analysis.

Method II [10] sorts genes based on the *P*-value for a regularized *t*-statistic calculated from each gene. Information from other genes plays no role in calculating the *P*-value for the current gene of interest. This, combined with the subjectiveness of the cutting rule for gene selection, may largely explains the difference between Method II and the proposed new method.

Method III [14] uses a *t*-statistic as the data point for cluster analysis. The original gene expression profile is converted into the *t* score. Some information may be lost during the conversion because the method fails to incorporate the error for calculating the *t* score. In addition, the *t* score is the differential gene expression divided by the standard deviation of the difference. However, the calculated standard deviation is subject to large error when the number of replicates per condition is small, which is usually the case in microarray experiments.

Similar to Method II, Method V [13] creates a list of significant genes based on the moderated *t*-statistic. Extra information is borrowed, on the basis of the hierarchical model, from the ensemble of genes which can assist in inference about each gene individually. However, users need to subjectively specify the mixing proportions before the algorithm is applied. The authors pointed out that the estimations of mixing proportions are somewhat unstable and suggested using 0.01 as a universal prior. In real mice data, the proportion of differentially expressed genes is about 0.002, which is overestimated by limma. This may explain why limma detected too many false positives both in simulation study and mice data analysis.

Method VI [15] is quite similar to the proposed method except that the observed differential expression between the control and the treatment is used for cluster analysis instead of using the expected differential expression (parameter) as the basis for clustering. Again, the error structure of the expression profile is not properly incorporated into the model when the normalization-like procedure is used. This explains why the proposed new method outperforms Method VI. In addition, Method VI is based on the EM algorithm, while the proposed method is based on the MCMC algorithm. The EM algorithm sometimes may stuck in the so-called local optimal, while the MCMC has reasonable chance to jump out of the “trap.” This may explain why Method VI failed in the situation where the borders of clusters are fuzzy with the effects of differentially expressed genes varying within a wide span, rather than a narrow span (data not shown).

In a quantitative trait-associated microarray data analysis, Jia and Xu [25] classified genes into several clusters based on the association of gene expression and the phenotypic value of a quantitative trait. The Bayesian method developed here can be used for such a quantitative trait-associated microarray data analysis. Recall that, in the differential expression analysis, the design matrix *X* is a variable indicating whether a gene comes from the control or the treatment. To make such an extension, the design matrix *X* in the differential expression analysis is simply replaced by the phenotypic values of the trait in the quantitative trait-associated microarray analysis. The method developed here has the following extra features compared to that of Jia and Xu [25]: (a) Bayesian method implemented via the MCMC algorithm, (b) constraints on the cluster means, (c) an imbedded normalization step, and (d) a fixed number of clusters.

In differential gene expression analysis, we usually deal with two conditions, control and treatment, with a purpose of identifying genes whose expression levels have altered between the two conditions. In time-course and dose-response [26] microarray experiments, however, gene expression are measured from multiple conditions. The Bayesian mixture model analysis developed here may be extended to detect differentially expressed genes across multiple conditions. To make such an extension, the dimensions of *X* and *γ_i_* in model (2) need to be changed to reflect the multiple conditions. Let *d* + 1 be the number of conditions. The modified dimensions of *X* and *γ_i_* should be *N* × *d* and *d* × 1, respectively. The change of *γ_i_* from a scaler into a vector leads to the following consequences: (1) the clusters of down- and upregulated genes are not explicitly defined, although the neutral cluster is still defined as that with *β_k_* = 0, and (2) the variance of *γ_i_* in the experiment with two conditions becomes a variance-covariance matrix in the experiment with multiple conditions. The first problem may be solved via the following approaches. Let *C* be the total number of clusters from which these genes are sampled. Let the first cluster be the one consisting of all the neutral genes and thus *β*_1_ = 0 is enforced for this cluster. The means of the remaining clusters *β*_2_ − *β*_C_ are not restricted, that is, they are sampled freely from their posterior distributions (multivariate normal). The number of clusters *C* are found based on the value of Bayesian factor or BIC. Genes not classified into the neutral cluster are claimed to be differentially expressed. The actual number of neutral genes may not be sensitive to the choice of the total number of clusters. Therefore, one may simply choose any reasonable number of clusters for the analysis. This number may be a function of *d*, say *C* = 3^*d*^. Note that *C* = 3 for a single dimension (one regression coefficient) as done in this study. For two regression coefficients, *C* = 3 × 3 = 9. Therefore, for *d* regression coefficients, the number of clusters becomes *C* = 3^*d*^. The second problem may be tackled as follows. The scaled inverse chi-square distribution chosen for the variance may be replaced by the generalization of the scaled inverse chi-square distribution for the variance-covariance matrix, called the inverse-Wishart distribution [21]. This prior distribution for the variance-covariance matrix is conjugate, and thus the standard sampling algorithm for a random vector from an inverse-Wishart distribution applies.

The intercept *α_i_* of gene *i* in model (2) is irrelevant to the differential expression analysis and thus it is removed from the model via a special normalization process, called linear contrasting. In fact, all effects not related to differential expression can be removed via such a normalization process. Deleting these irrelevant effects (e.g., dye effect, block effect, etc.) may avoid tedious estimating procedure for unspecified parameters used in Zhang et al. [1] and save substantial time in calculation. To remove these effects, matrices *W* and *α_i_* need to be customized to reflect the general nature of the normalization. Let *h* be the number of irrelevant effects. The dimensions of *W* and *α_i_* should be *N* × *h* and *h* × 1, respectively. The coefficient matrix for the linear contrasts *L* remains the same as that defined by model (6). However, matrix L* takes the first *N* − *r*(*W*) eigenvectors of matrix *L*. This generalized approach of normalization is conceptually similar to the ANOVA analysis proposed by Kerr et al. [27], where two steps are involved in the ANOVA. In the first step,*α_i_* is estimated under model $Y_{i}=W\alpha_{i}+\epsilon_{i}$ and then $Y_{i}^{*}=Y_{i}-W{\hat{\alpha }}_{i}$ , the residuals adjusted by the irrelevant effects, is used as the raw data for further differential expression analysis. In the second step, *Y_i_* * is described by model $Y_{i}^{*}=X\gamma_{i}+\epsilon_{i}$ and then *γ_i_* is estimated and tested to make an inference about the significance of gene *i*. Three issues need to be emphasized for the comparison: (1) the generalized approach of normalization proposed in this study removes *α_i_* using no explicit estimate of *α_i_*, (2) the reduced degrees of freedom after adjusting for the irrelevant effects are used for the new method, and (3) appropriate covariance structure for the residuals is used for the new method.

## 5. CONCLUSIONS 

In this paper, we propose a Bayesian mixture model approach to detect genes that differentially expressed in control-treatment microarray experiments. Genes are assigned into one of three constrained clusters, corresponding to clusters of downregulated, neutral, and upregulated genes, respectively. The Bayes method is implemented via the Markov chain Monte Carlo (MCMC) algorithm. Genes that have been assigned into nonneutral clusters are the target genes which we would like to disclose. Using simulated data as well as data from real microarray experiments, we demonstrate that the new method outperforms the methods commonly used in differential expression analysis. Although the new method was demonstrated using data generated from laboratory animals, it is able to generalize to genome studies for plants.

## Figures and Tables

**Table 1:** Parameters used in the simulation experiment and their estimates from the Bayesian mixture model analysis.

Parameter		*α_k_*	*β_k_*	*π_k_*	*ν_k_*	*σ* ^2^
True	Group 1	3.5	1.2	0.005	0.01	0.03
	Group 2	4	0	0.345	0.04	
	Group 3	4.5	−1	0.02	0.02	
	Group 4	0	0	0.615	0.04	
	Group 5	0	0.8	0.005	0.01	
	Group 6	0	0.9	0.01	0.01	

True (combined)	Cluster 1	—	−1	0.020	0.02	0.03
	Cluster 2	—	0	0.960	0.04	
	Cluster 3	—	0.95	0.020	0.03	

Estimated	Cluster 1	—	−0.984	0.023	0.03	0.03
	Cluster 2	—	0	0.937	0.04	
	Cluster 3	—	0.702	0.040	0.11	

**Table 2:** Numbers of genes assigned into each of the three clusters for six different methods of differential expression analysis. (The sum of each column within a method represents the true number of genes simulated from that cluster and the sum of each row represents the number of genes assigned into that cluster.)

Method	Estimate	True			Sum	Type I error
		Cluster 1	Cluster 2	Cluster 3		
I	Cluster 1	20	1	0	21	0.002
	Cluster 2	0	958	0	958	
	Cluster 3	0	1	20	21	

II	Cluster 1	20	205	0	225	0.459
	Cluster 2	0	519	0	519	
	Cluster 3	0	236	20	256	

III	Cluster 1	0	0	0	0	0.033
	Cluster 2	0	928	0	928	
	Cluster 3	20	32	20	72	

IV	Cluster 1	20	37	0	57	0.073
	Cluster 2	0	890	0	890	
	Cluster 3	0	33	20	53	

V	Cluster 1	20	153	0	173	0.325
	Cluster 2	0	648	0	648	
	Cluster 3	0	159	20	179	

VI	Cluster 1	20	5	0	25	0.011
	Cluster 2	0	949	0	949	
	Cluster 3	0	6	20	26	

	Sum (method)	20	960	20	1000	

**Table 3:** Parameters estimated for the mice data using the Bayesian mixture model analysis.

	*β_k_*	*π_k_*	*ν_k_*	*σ* ^2^
Cluster 1	−0.0065	0.1724	0.0616	0.0904
Cluster 2	0	0.8246	0.0006	
Cluster 3	0.6418	0.0030	2.0266	

**Table 4:** Numbers of genes assigned to the three clusters for the mice data for six different methods.

Method	Cluster 1	Cluster 2	Cluster 3
	(down)	(neutral)	(up)
I	0	6332	10
II	40	6182	120
III	66	6276	0
IV	13	6300	29
V	12	6293	37
VI	0	6329	13

**Table 5:** Some differentially expressed genes for the mice data detected by six different methods.

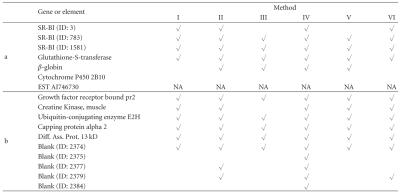

a: genes reported in Callow et al. [23]; b: a subset of genes not reported in Callow et al. [23].

## References

[1] Zhang D, Wells MT, Smart CD, Fry WE (2005). Bayesian normalization and identification for differential gene expression data. *Journal of Computational Biology*.

[2] Jörnsten R, Wang H-Y, Welsh WJ, Ouyang M (2005). DNA microarray data imputation and significance analysis of differential expression. *Bioinformatics*.

[3] Efron B, Tibshirani R, Storey JD, Tusher V (2001). Empirical bayes analysis of a microarray experiment. *Journal of the American Statistical Association*.

[4] Broët P, Richardson S, Radvanyi F (2002). Bayesian hierarchical model for identifying changes in gene expression from microarray experiments. *Journal of Computational Biology*.

[5] Edwards JW, Page GP, Gadbury G (2005). Empirical Bayes estimation of gene-specific effects in micro-array research. *Functional and Integrative Genomics*.

[6] Do K-A, Müller P, Tang F (2005). A Bayesian mixture model for differential gene expression. *Journal of the Royal Statistical Society. Series C*.

[7] Medvedovic M, Sivaganesan S (2002). Bayesian infinite mixture model based clustering of gene expression profiles. *Bioinformatics*.

[8] Vogl C, Sanchez-Cabo F, Stocker G, Hubbard S, Wolkenhauer O, Trajanoski Z (2005). A fully bayesian model to cluster gene-expression profiles. *Bioinformatics*.

[9] Teschendorff AE, Wang Y, Barbosa-Morais NL, Brenton JD, Caldas C (2005). A variational Bayesian mixture modelling framework for cluster analysis of gene-expression data. *Bioinformatics*.

[10] Tusher VG, Tibshirani R, Chu G (2001). Significance analysis of microarrays applied to the ionizing radiation response. *Proceedings of the National Academy of Sciences of the United States of America*.

[11] Dudoit S, Yang YH, Callow MJ, Speed TP (2002). Statistical methods for identifying differentially expressed genes in replicated cDNA microarray experiments. *Statistica Sinica*.

[12] Cui X, Churchill GA (2003). Statistical tests for differential expression in cDNA microarray experiments. *Genome Biology*.

[13] Smyth GK (2004). Linear models and empirical bayes methods for assessing differential expression in microarray experiments. *Statistical Applications in Genetics and Molecular Biology*.

[14] Pan W (2002). A comparative review of statistical methods for discovering differentially expressed genes in replicated microarray experiments. *Bioinformatics*.

[15] Gusnanto A, Ploner A, Pawitan Y (2005). Fold-change estimation of differentially expressed genes using mixture mixed-model. *Statistical Applications in Genetics and Molecular Biology*.

[16] Newton MA, Noueiry A, Sarkar D, Ahlquist P (2004). Detecting differential gene expression with a semiparametric hierarchical mixture method. *Biostatistics*.

[17] Dempster AP, Laird NM, Rubin DB (1977). Maximum likelihood from incomplete data via the em algorithm. *Journal of the Royal Statistical Society: Series B*.

[18] Benjamini Y, Liu W (1999). A step-down multiple hypotheses testing procedure that controls the false discovery rate under independence. *Journal of Statistical Planning and Inference*.

[19] Patterson HD, Thompson R (1971). Recovery of inter-block information when block sizes are unequal. *Biometrika*.

[20] Devroye L (1986). *Non-Uniform Random Variate Generation*.

[21] Gelman A, Carlin JB, Stern HS, Rubin DB (1995). *Bayesian Data Analysis*.

[22] Schwartz G (1978). Estimating the dimensions of a model. *The Annals of Statistics*.

[23] Callow MJ, Dudoit S, Gong EL, Speed TP, Rubin EM (2000). Microarray expression profiling identifies genes with altered expression in HDL-deficient
mice. *Genome Research*.

[24] Westfall PH, Young SS (1993). *Resampling-Based Multiple Testing: Examples and Methods for P-Value Adjustment*.

[25] Jia Z, Xu S (2005). Clustering expressed genes on the basis of their association with a quantitative phenotype. *Genetical Research*.

[26] Peddada SD, Lobenhofer EK, Li L, Afshari CA, Weinberg CR, Umbach DM (2003). Gene selection and clustering for time-course and dose-response microarray experiments using order-restricted inference. *Bioinformatics*.

[27] Kerr MK, Martin M, Churchill GA (2001). Analysis of variance for gene expression microarray data. *Journal of Computational Biology*.

